# “How Do You Feel about Antibiotics for This?” A Qualitative Study of Physician Attitudes towards a Context-Rich Communication Skills Method

**DOI:** 10.3390/antibiotics2030439

**Published:** 2013-09-16

**Authors:** Jochen W. L. Cals, Mirjam E. van Leeuwen, Fleur H. F. Chappin, Eefje G. P. M. de Bont, Geert-Jan Dinant, Christopher C. Butler

**Affiliations:** 1 Department of General Practice, School for Public Health and Primary Care (CAPHRI), Maastricht University Medical Centre, PO Box 616, 6200 MD Maastricht, The Netherlands; E-Mails: mirjam.vanleeuwen8@gmail.com (M.E.L.); fleurchappin@ziggo.nl (F.H.F.C.); eefje.debont@maastrichtuniversity.nl (E.G.P.M.B.); geertjan.dinant@maastrichtuniversity.nl (G.-J.D.); 2 Institute of Primary Care and Public Health, School of Medicine, Cardiff University, Cardiff CF14 4YS, UK; E-Mail: butlercc@cardiff.ac.uk

**Keywords:** qualitative study, communication training method, patient-centered, primary care, communication items, LRTI

## Abstract

To explore experiences with and views of general practitioners (GPs) on a physician communication training method in primary care and its applicability and implementation in daily practice, we performed a semi-structured qualitative study of GPs’ experience of training in and implementing a communication skills training program for managing lower respiratory tract infection (LRTI) which included a seminar, simulated patient consultation together with providing and receiving feedback on ones own transcript, and a seminar in a structured approach to the LRTI consultation. Seventeen out of 20 eligible GPs who had participated in the IMPAC^3^T trial and were allocated to receiving enhanced physician communication training for managing lower respiratory tract infection participated. GPs’ experiences with the physician communication training method and its specific components were positive. The method gave GPs additional tools for managing LRTI consultations and increased their sense of providing evidence-based management. During the study, GPs reported using almost all communication items covered in the training, but some GPs stated that the communication skills diluted over time, and that they continued to use a selected set of the skills. The general communication items were most regularly used. Implementation of the method in daily practice helped GPs to prescribe fewer antibiotics in LRTI with the only perceived disadvantage being time-pressure. This study suggests that GPs felt positive about the physician communication training method for enhanced management of LRTI in primary care. GPs continued to use some of the communication items, of which general communication items were the most common. Furthermore, GPs believed that implementation of the communication skills in daily practice helped them to prescribe fewer antibiotics. The context-rich communication method could have wider application in common conditions in primary care.

## 1. Introduction

The quality of doctor-patient communication is crucial in general medical practice. Models in patient-doctor communication set out to make the implicit in patient care explicit. However, while models help to clarify the basics in communication, they never completely capture what happens in reality. With patient-centered care being heavily promoted, patient-centered clinical methods need to be developed and taught [1]. Implementation and assessment of acquired communication skills is challenging. Competence assessments measure what doctors can do in controlled representations of professional practice; performance assessments measure what doctors do in actual professional practice. Often, only competence is measured as these assessments are generally easier administered through exams or structured observations. However, the perspectives of patients and society demand that doctors should meet the assessment standards in their working conditions in any given situation. It has therefore been suggested that the emphasis in assessing communication skills should lie on the assessment of performance [2].

Physicians in primary care are faced with consultations involving antibiotic prescribing decisions on a daily basis and these consultations are often difficult due to a variety of factors [3,4]. We developed an innovative, largely practice-based and context-rich communication skills method for general practitioners (GPs), for the exemplar condition of lower respiratory tract infection (LRTI) [5]. LRTI in particular is one of the most frequently encountered illness in general practice, and there is substantial antibiotic over prescribing for the condition that is influenced by an array of clinical and non clinical factors [6,7]. GPs need sophisticated communication skills to manage non-medical influences within the consultation. Furthermore, possible benefits and harms of antibiotic treatment need to be explored, while responding to the patient’s agenda and setting realistic expectation about usual illness course and when to re-consult. 

Key features of the training were its context-rich nature, working with simulated patients [8], the innovative use of “peer-review” of colleague’s transcripts with simulation patient (SP) and the brevity of the workshop-based training, with a total time investment for the full program of 4 h. We previously demonstrated a significant increase of evidence in competence in selected core communication skills for managing LRTI in primary care [5]. Moreover, GPs allocated to receiving the communication skills training prescribed significantly fewer antibiotics in our trial-based assessment of performance of the enhanced communication skills training [9] and patients were less likely to receive antibiotics in the years after being exposed to a GP using these communication skills [10].

We therefore aimed to explore GPs’ experiences in the training they received and in using the method and its specific components in daily practice, including system- and personal-related influences [2]. We hoped that this might not only enhance the training and the intervention itself, but that it might also facilitate the application of (components) of this method in communication strategies for managing a range of other conditions in primary care.

## 2. Methods

### 2.1. Subjects

We aimed to interview the 20 general practitioners from 10 general practices in the South-eastern part of The Netherlands who were involved in this aspect of the original trial. These GPs were part of the IMPAC^3^T study (ISRCTN85154857), which recruited patients from autumn 2005 until spring 2007. IMPAC3T was a factorial, cluster randomized clinical trial assessing the effect of a enhanced physician communication training, singly and combined with a near patient C-reactive protein (CRP) test, on antibiotic prescribing for LRTI [11]. In this trial, 20 GPs were allocated to receiving enhanced physician communication training for LRTI. Ten of these GPs were exposed to the novel communication training method additionally received training in the near patient test for LRTI. However, this was a separate intervention and is not part of this interview study. The different components of the communication training method were: simulated patient encounter in daily clinical practice, a brief workshop using transcripts of those encounters, another simulated patient encounter in the own surgery during regular consultation hours, and “peer-review” by two colleague’s of the transcripts of that simulation patient encounter. A full description of the method and analysis of GPs’ competence in implementing the acquired skills in daily practice after training have been reported previously [5]. The trial results [9], long term outcomes [10], and GPs’ attitudes towards the point of care test [12] have been described elsewhere. All GPs provided written consent to participate in our study. Ethical approval was obtained by the Ethics Committee of Catharina Hospital in Eindhoven, The Netherlands. 

### 2.2. Interview Procedure

We chose a qualitative study design, as our goal was to explore respondent’s beliefs and understanding of the experiences and opinions of the GPs. Two trained interviewers (ML and FC) conducted semi-structured interviews, using an interview guide to structure the interviews with an average length of 30 min per GP. The interviews were practice-based, audio-taped and took place in the first winter after patient recruitment for the trial came to an end, which was 8 months at most. The GPs were told that our purpose was not to audit practice but to understand their experiences and views about the communication training method implemented in the trial. 

The interview guide was pilot-tested through one video-taped consultation with a GP from our study group. Interviews included questions about the specific components of the communication training method, advantages and disadvantages of the method and its components, experiences of implementing the communication items addressed during the training in daily practice, and whether or not the communication method affected their antibiotic prescribing, also beyond LRTI. All questions were open, followed by prompts when there was no response to initial questions. We added new sub-items to main themes in the topic list as the interview process progressed and new insights emerged. Theoretical data saturation was defined as no new themes emerging, in line with grounded theory [13]. Data saturation was recorded, yet we aimed to interview all pre-defined 20 GPs. 

### 2.3. Data Analysis

The audio-taped interviews were anonymized and transcribed by an experienced medical typist. The transcripts were used as a basis for analysis and were read triple, by ML, FC and JC. Analysis and data collection were conducted in parallel and was assisted by NVivo software. Coding schedules were agreed and piloted. Reliability was assured by coding 70% of the interviews by more than one researcher (ML or FC). Discrepancies were resolved by discussion. In case of disagreement, a third decisive rater (JC) was consulted. We sought to identify commonly expressed themes as well as unusual cases. A thematic content analysis was conducted on GPs’ responses. This method of analysis is essentially a process of summarization, categorization and counting frequency of responses [13].

## 3. Results

### 3.1. Subjects

Seventeen of the 20 eligible GPs were interviewed. One GP declined without reason, two GPs had ceased practice and could not be contacted. GPs had an average age of 48.8 years (range 36–59 years), and 7 were female. No new items emerged after 12 interviews, but the all 17 predefined interviews were completed and analyzed. 

### 3.2. Attitudes towards the Full Programme

Most practitioners mentioned that the training seminar had given them additional tools for LRTI consultations, especially in giving appropriate information and explanation to patients. For a few doctors, the main strength of the training method was that the training seminar refreshed their communication skills learnt during undergraduate medical education: “*I believe that the strength of repetition is used. Many things corresponded to what we already knew, but were reinforced*” *(GP13).* Another commonly cited strength of the training seminar was the consultation with simulated patient: “*Obviously, the simulation patient before and after the training is a strong aspect, this provides a unique learning opportunity*” *(GP2).* For some GPs, the strength lay in the structured approach to LRTI consultations and the opportunity to reflect on their communication skills. A strong aspect mentioned by most GPs was the practice based setting of the learning opportunity, as they found this time-efficient, convenient, more authentic, and more satisfying. 

The majority of the practitioners could not identify any limitations of the communication training method. Only a few suggested that real patients would be superior to simulated patients in practicing skills “*If you would really want to improve the training and get a better understanding on how things go, you would have to replace simulation patients by actual patients and instruct them in advance*” *(GP14).* Other limitations included the wish for a more intensive training, the longer time-delay between the simulated patient contact and the connected opportunity to provide peer-review on that transcript. However, these limitations were only mentioned once. 

### 3.3. Experience with the Specific Training Components

#### 3.3.1. Simulated Patients

Most practitioners said they enjoyed working with simulated patients and they believed it was a positive, pleasant and a useful experience. “*I think it’s very useful. I believe it makes you more alert and willing to perform well*” *(GP3).* Some practitioners thought the consultation was unnatural and made them feel insecure. Some said the consultation situation, although practice-based, was not comparable with their everyday practice and several GPs felt the consultation was slightly artificial. 

#### 3.3.2. Communication Skills Training Seminar

All but two practitioners felt positive about the training seminar. Some mentioned it was “instructive” and “useful”, especially the tools provided to improve LRTI consultations. Some practitioners mentioned it was a good opportunity to improve their communication skills in general: “*I considered it a very positive experience, it was an opportunity to practice and you were handed actual tools like applicable phrases on how to discuss things with patients. I believe this to be very important*” *(GP1).* Others said it created awareness about their LRTI routine practice. A few said they particularly enjoyed sharing information on dealing with LRTI consultations “*I also enjoyed hearing from others how they handled things at that moment*” *(GP8).* Two doctors thought the training seminar was redundant because of their broad clinical experience. 

#### 3.3.3. Transcripts

In general, all GPs were positive about using transcripts during the seminar and they enjoyed reading them. Several practitioners mentioned that reading their transcript was challenging because of their shortcomings: “*Yes it is always very confronting to see what you do and do not ask, things you forget to ask, even shortly after this kind of training*” *(GP2).* They also believed it was instructive to have an overview of their way of practicing “*Many times you believe to do something, while in fact you just don’t, therefore the training was an eye-opener and a pleasant experience*” *(GP11)* However, the absence of intonation and non-verbal communication between GP and simulated patient were mentioned as a disadvantage by some clinicians. 

#### 3.3.4. Peer-Review

The absence of nonverbal communication was also mentioned as a disadvantage when peer-reviewing a colleagues transcript: Nonetheless, the majority of the doctors enjoyed the peer-review process, especially as some stated that it enables one to see how colleagues proceed in LRTI consultations. Some experienced difficulty in giving negative feedback to their colleague. A striking quote was: “*Reading the feedback [of a colleague] was actually almost more enjoyable than patients in daily practice*” *(GP6).* Two practitioners could not recall this aspect of the method as a result of the time gap. 

### 3.4. Past, Present and Future Use of the Communication Skills

In general, the application of the communication items varied between the participated doctors. The majority said that in the beginning they used almost all communication items. Currently, GPs still regularly use some of the communication items as one doctor explained: “*Checking whether or not a patient understands what I have said. I most certainly do that. Before someone leaves I actually try to figure out whether or not we discussed the patients’ expectations and if they received advice they can use*” *(GP8).*

Strikingly, the general communication items were most regularly used “All of the general communication items. Because of the fact that those are the items which belong in a proper consultation” (GP3). It was mentioned that these skills were already stressed at medical school. The majority of the doctors said they particularly try to elicit expectations about management. “Checking patients understanding of the given information” and “reaching agreement with the patient on proposed treatment”, are also frequently used by about half of the doctors. Half of the practitioners thought that “summarizing the consultation” was unnecessary and others admitted to forget to summarize: “Summarizing remains difficult. Apparently I never do it. I see it written down now and think to myself: ‘I never remember doing that’” (GP5). 

The most frequently used LRTI specific communication item during the study period was “actively ask for patient’s opinion on antibiotics”. One practitioner said: “*Especially the question what patients think about antibiotics. Most of the time you decide about the necessity for antibiotics without checking the patients needs. If you do check this you can better correspond to the patients expectations*” *(GP3).* This item was often mentioned, exemplified by these quotes: “*Talking about their opinion on antibiotics created an opportunity to speak about it without attending the discussion whether or not antibiotics are appropriate in this case. Also, pointing out that coughing can go on for a while. Yes, I believe that for most patients this can be an eye-opener*” *(GP13)* and “*The thing I found enlightening was asking for the patient’s opinion. At first it felt strange, but by doing so you discover that there are many patients who do not necessary want antibiotics. And this is something I didn’t do in advance*” *(GP1).* Most GPs said that they always mentioned a likely duration of cough before the study period and continued doing so. Furthermore, “mentioning the pros and cons of antibiotic treatment to the patient” were used by the majority of the doctors during the study and they intent to continue using that skill as they feel it gives them the tool to convince patients about antibiotic treatment. A typical clinician’s quote was: “*you are capable of solving this yourself without antibiotics. Sometimes they jumped up as if they were saying “O yes that’s right!”*” *(GP4).*

About half of the practitioners considered repeating the communication training to be useful and they stressed out the importance of communication skills in general “*The most important tool a GP—and every other doctor for that matter—has, is communication. I believe that when you optimize this tool and stimulate others to do so, you improve your tools as a professional. Because of this I believe it is a sensible thing to do*” *(GP11).* Most other GPs said that repetition would not be useful. However, a few did admit that the use of specific skills diluted over time. Several practitioners mentioned they would prefer to undergo a similar training for other conditions, preferably in a broader spectrum of respiratory illnesses. 

GPs mentioned that the ideal frequency for refresher training was from twice a year to once every five years. Several doctors said they wanted to repeat the simulated patient contacts. Others wanted to repeat the training seminar and a few would like to get the chance to practice with the communication skills concurrently. GPs mentioned they would be more motivated to attend in the communication training method if CME points would be awarded.

### 3.5. Influence of the Method on Daily Practice and Antibiotic Prescribing Decisions

In general, almost all GPs were positive about the implementation of the communication skills in daily practice. Most practitioners mentioned they had a more evidence-based management and were more aware of their own way of practice, and they felt it helped smoothing their routine in LRTI consultations. A few practitioners mentioned that it changed their consultations: “*perhaps I usually use these types of consultations to make up for lost time. Now they have become more of a challenge, enjoyable and the conversations with people changed because of this*” *(GP5).* Several felt more able to achieve a shared evidence based antibiotic prescribing decision. A minority recognized that they had some form of prejudice about patients’ expectations and they felt this decreased by actively eliciting patients’ expectations about management. 

A few GPs said the most important prognostic skill they had acquired was the communication skills, as this gave them tools for providing a better explanation to patients about their decisions, and as a result patients were more willing to accept the self-management strategy. “*That you shouldn’t talk about viruses and bacteria, but that you should clearly state, it is an infection and your body is capable of resolving it by itself. I thought that was really the key*” *(GP4).* In addition, the GPs felt more confident about their explanation and their proposed policy, “*You are more confident as a general practitioner towards your patient because of the fact that you have more tools to justify a wait-and-see policy. Although you know for yourself this is the best way, you also have to convince the patient*” *(GP1).* Furthermore, a few practitioners said they had a guideline and therefore a better construction for LRTI consultations. Others felt they were able to improve the quality of their consultations in general. Some doctors mentioned it was easier to identify the patient’s agenda and patient’s expectations and it was easier to comfort them. “*As an obvious addition for the structure of the consultation, addressing someone’s concerns, and as a tool to tell the story in a way the patient finds reassurance*” *(GP12).* Some felt through the communication skills their policy was evidence-based, and patients were more willing to accept their explanation. A few said they needed less time “coming to the point”, because they could efficiently identify the patient’s *agenda:* “*I believe you find out sooner what the underlying reason of a patients visit is. People with a cough actually have a lot of thoughts about this*” *(GP10).* Several GPs thought the communication skills were applicable in all consultations, especially in consultations concerning antibiotic prescribing like sore throat or cystitis: “*I think there are no downsides to addressing something with a systematic approach. The advantage is that you can apply communication skills in different settings. It is obviously not limited to respiratory infections*” *(GP12).*

About half of the GPs didn’t report experiencing any disadvantages of implementing the skills in daily practice. The other half said that in case of time-pressure, practitioners have the tendency to skip the talking. However, some mentioned that the extra time was a good investment for following LRTI consultations and it is not a disadvantage at all.

The majority of the GPs believed they prescribed fewer antibiotics to their LRTI patients as a result of the communication skills training “*Because I am better at reassuring and probably give a clearer explanation on how they can deal with their complaints, it think I prescribe fewer antibiotics*” *(GP12).*

## 4. Discussion

### 4.1. Main Findings

We achieved a deeper understanding of GPs’ experiences with and attitudes towards a newly developed physician communication training method for the exemplary condition of LRTI. In general, GPs experiences with the physician communication training method, and its specific components, were positive. The method had given them additional tools for managing LRTI consultations, leading to more evidence-based management. While in the beginning, almost all communication items were used, the range of skills they deployed diminished over time. Implementing the communication skills learnt through the training method in daily practice helped the GPs to prescribe fewer antibiotics for LRTI and the only reported disadvantage of the communication skills in practice was that is could add to time pressures in consultations. 

### 4.2. Comparison with Other Studies

This communication skills training had the desired effect on the pre-defined outcome measure (antibiotic prescribing), yet an understanding of experiences with and attitudes towards the training method itself is crucial in explaining the observed effect and in exploring future applications of the method itself. Specialist training in general practice is a big challenge. Once physicians are well established in clinical practice, they rarely receive specific training to enhance their communication skills [14]. Additionally, patient-centeredness and communication skills do not have such an important position in educational programs. Most undergraduate programs in The Netherlands do involve some extent of communication skills training. In our study, we aimed to explore GPs views about repeating communication skills in educational programs, and half of the GPs responded positive. 

Most studies focused on changing physicians’ communication skills were only effective in short-term [15]. We previously showed this communication training method was effective in changing GPs consulting behavior in short-term and long-term as well [5]. Other primary care studies based on the same principle of health behavior counseling [16,17] were all highly successful in decreasing inappropriate prescribing for infections in primary care [18,19]. In another study, patient-centered communication training did not reduce the rate of antibiotic prescriptions below an already low level [20].

### 4.3. Strengths and Limitations

One of the strengths of our study is the qualitative study design. Qualitative research is mainly exploratory and analytical, dealing with research questions about exploring beliefs, understandings or cultures and it produces findings not by statistical procedures or other means of quantification [21]. We chose semi-structured interviews because they are conducted on the basis of a loose structure consisting of open ended questions that define the area to be explored and from which the interviewer or interviewee may diverge in order to pursue an idea in more detail [21]. Structured interviews consist of administering structured questionnaires, and interviewers are trained to ask questions in a standard manner and in depth interviews are less structured and may cover only one or two issues [21]. Therefore, these two types of interviews were less appropriate for our study.

Since we successfully interviewed 17 out of 20 eligible practitioners, we are confident we covered all important data. In qualitative research, a sample size is not determined by hard and fast rules, but by other factors such as the depth and duration of the interview and what is feasible for a single interviewer [21]. In addition, all interviews were audio-taped and transcribed instead of writing notes during or after the interview. It did not interfere with the interview process, further enhancing the quality of our data. We obtained coding reliability by double coding the majority of the transcripts. We did not stop data collection after data saturation had been reached, since we aimed to interview all predefined 20 GPs. Recall bias may be an issue as there was an eight month time gap between the end of the trial and the interviews. Some GPs could not recall information about various topics, therefore some interviews were brief and this could possibly interfere with the results. 

GPs were unaware of the results, *i.e.*, the effectiveness, of the trial interventions, since the report was not published at the time the interviews took place [9,10]. As a result, this could not have interfered with GPs expectations and views on the physician communication training method. From previous studies, we know that physicians’ and patients’ perspectives on antibiotics may differ substantially [6,22]. We did not take patients’ satisfaction or attitudes towards receiving this kind of consultation into account. This is a relevant topic for future qualitative research.

## 5. Conclusions

Our results may guide investment in primary care to the communication training method. Management in a wider range than just LRTI could be considered, since GPs mentioned the communication skills could be used for most common infections in primary care. Nevertheless, implementation of the communication training method on a larger scale could be difficult because of various organizational restrictions, *i.e.*, the number of simulated patients needed, training seminars should be given in more than one location in The Netherlands to decrease travel time. Alternatively, a less intensive method by providing training online could be an option, or a repetition of the method combined with the emergence of new guidelines [23]. However, the effects of such an approach are unknown. Moreover, the perceived time investment within the consultation could be a barrier for continuous use in daily practice.

This study suggests that GPs felt positive about an innovative physician communication training method using practice-based contacts with simulated patients, peer-review of transcripts and an interactive seminar reflecting on these consultations to optimize their acute cough consultations and antibiotic prescribing decisions. They felt that the program and its specific components gave them additional tools and a more evidence-based management in LRTI consultations. 
